# COVID-19 Vaccination Status and Attitudes of Family Child Care Providers in Delaware, September 2021

**DOI:** 10.3390/vaccines10030477

**Published:** 2022-03-19

**Authors:** Laura Lessard, Rena Hallam, Deborah Drain, Laurie Ruggiero

**Affiliations:** 1 Department of Behavioral Health and Nutrition, University of Delaware, Newark, DE 19711, USA; ruggiero@udel.edu; 2 Department of Human Development and Family Sciences, University of Delaware, Newark, DE 19711, USA; rhallam@udel.edu (R.H.); ddrain@udel.edu (D.D.)

**Keywords:** COVID-19, vaccination, child care

## Abstract

Child care providers, including family child care (FCC) providers, are viewed as trusted sources of information for the parents and families they serve, and their vaccine behavior has been shown to be associated with parent beliefs and behaviors. This study sought to describe the COVID-19 vaccine behaviors and attitudes among FCC providers in Delaware. An online survey was distributed to all licensed FCC providers (*N* = 541) in September 2021. Survey items were drawn from validated instruments and assessed vaccination status, attitudes, and confidence in their ability to discuss COVID-19 vaccines with families. In total, 168 responses were recorded (31% response rate); 69.8% of respondents were fully vaccinated against COVID-19. The majority indicated that they would require (11.4%) or recommend (42.1%) the vaccine for children in their care, once approved by the FDA. Providers reported high levels of confidence in their ability to discuss both the benefits and risks of COVID-19 vaccines. FCC providers should be considered key messengers for the families they serve and may be helpful liaisons with state and local vaccination efforts. Input from FCC providers could be used to develop vaccine messaging and materials that are clear, consistent, and culturally responsive to the families of the children in their care.

## 1. Introduction

The COVID-19 pandemic has impacted early care and education (ECE) settings across the globe, including center-based programs where same-age children are watched in institutional settings and family child care programs, where a small group of mixed age children are cared for in a home setting. In the early stages of the pandemic, many ECE programs were forced to close or opted to close to protect the health and safety of the children and the ECE workforce. For example, one study estimated that 35% of center-based and 21% of family child care programs in the United States (US) were closed during summer 2020 [1]. Furthermore, for the programs that remained open, enrollment was estimated to be down by 67% [2].

To facilitate the reopening of ECE programs, public health officials released guidance on how these programs could safely operate during the pandemic, while minimizing transmission risk within programs. The guidance from the US Centers for Disease Control and Prevention (CDC) highlighted the benefits of a layered approach to mitigation, including universal masking, social distancing, keeping children in small groups, and limiting enrollment [3]. As COVID-19 vaccines became available, ECE providers were among the highest priority populations for access [4] and the CDC added ECE staff vaccination to the list of recommended mitigation practices. In Delaware, child care providers became eligible for COVID vaccination on 19 January 2021 and special vaccine clinics were held for providers.

Despite the ubiquity of ECE programs that serve an estimated 60% of US children ages 0–5 [5], there is limited research on vaccine uptake and hesitancy among ECE providers. One national study on ECE providers conducted in May and June 2021 suggested that overall vaccination rates may be as high as 78%, but there is significant variation within the provider community. Providers of color, those with lower income, and family child care providers reported lower rates of COVID-19 vaccination compared to their peers [6]. Lower vaccination rates are not unusual for this population, as ECE providers have shown lower rates of influenza vaccination than the general population [7].

The Family Child Care (FCC) setting, where individuals care for small groups of mixed age children in a home setting, is among the most popular ECE choices for families in the United States. Among infants with at least one weekly nonparental care arrangement, 26% are in non-relative care settings like FCC [5]. Since infants cannot practice any of the COVID-19 risk mitigation strategies (e.g., masking, distancing), their risk is determined by the behaviors of other children and adults in their environment, including FCC providers.

The FCC workforce is primarily female, disproportionately women of color and generally low-income; the median hourly wage for home-based providers was USD 10.35 in 2017 [8]. This workforce has limited access to health insurance through their employment, few sick and vacation days, and strong physical demands from their caregiving activities [9]. Families with children in FCC programs rely on providers for child development and parenting information, and providers are viewed as a valued source of health information [10]. Parents trust that FCC providers are knowledgeable in how to care for their children in a way that ensures child health and safety. Similarly, FCC providers are aware of their role in supporting the health of the children in their care, but also their role in communicating health information to the families of the children that they serve [11,12]. Previous research suggests that child care provider attitudes towards vaccination are associated with parent beliefs and behavior [13].

Given their role as trusted care givers, FCC provider vaccination status and vaccination attitudes have the potential to impact the health of the children they serve along with the vaccination decisions of the families they serve. This study sought to understand the nature of vaccination behaviors and attitudes among FCC providers in Delaware.

## 2. Materials and Methods

Participants and Recruitment: Licensed FCC providers in Delaware were eligible to participate in the online survey. In order to recruit providers, a series of emails, including a description of the study and a link to the survey, were sent from the state child care licensing office to a list of licensed providers (approximate *N* = 541). The survey was open from 17 August 2021 through 25 September 2021. One initial email invitation and three email reminders were sent. Respondents were entered into a raffle to win one of 15 USD 200 gift cards. All study-related procedures were approved by the Institutional Review Board at the University of Delaware.

Instrument: The survey link navigated participants to an online survey on the Qualtrics platform. COVID-19 vaccination questions were included in a longer self-report survey on FCC provider well-being. Survey items were drawn or adapted from existing, validated questionnaires. Demographic and health status items were drawn from the Behavioral Risk Factor Surveillance System (BRFSS). Participants were asked “Have you received a COVID-19 vaccine?” and “Did you receive (or do you plan to receive) all required doses”, which were drawn from the Centers for Disease Control and Prevention’s (CDC) Vaccine Confidence Survey Questions Bank. Likert-type questions on vaccine barriers contained statements and asked respondents to identify their level of agreement (strongly disagree to strongly agree). These items were drawn from Wong et al. [14] (e.g., I am concerned about the efficacy of the COVID-19 vaccine. I am concerned about the safety of the COVID-19 vaccine) and the U.S. Census COVID-19 Household Pulse Survey [15] (e.g., I am concerned about possible side effects of a COVID-19 vaccine).

Data analysis: Data were cleaned to remove duplicates and respondents who completed less than 20% of the survey. Frequencies for each item were calculated, and relationships between categorical variables were calculated using Chi-square tests. Item level analyses excluded respondents who skipped applicable items.

## 3. Results

In total, 168 valid responses were recorded (31% response rate). About one-third (34.3%) of respondents were Black or African-American, 38.3% of respondents had one or more children living in their household, and 12.4% reported not having any kind of health insurance (see Table 1).

Respondent experiences related to COVID-19 indicated that 29% had pre-existing conditions that put them at higher risk and 8.9% had been diagnosed with COVID-19. A total of 69.8% of respondents reported being fully vaccinated, with 22.8% being unvaccinated. Among those who were vaccinated, the majority (59.3%) reported receiving their first dose prior to April 2021, indicating that they were vaccinated near the earliest opportunity. There was no relationship between vaccination status and FCC provider race (Chi-square *p* = 0.177) or health insurance status (Chi-square *p* = 0.282).

Among the 22% of FCC providers who were not vaccinated, 17.6% indicated that they did not intend to ever receive the vaccine; 11.8% planned to receive it as soon as possible; 20.6% intended to wait and see how it affected others before receiving it; and 41.2% did not intend to receive it soon but may in the future (8.8% undecided).

The majority of providers indicated that they would require (11.4%) or recommend (42.1%) the COVID vaccine for children in their care, once approved by the FDA. About one quarter of FCC providers were unsure what their policy approach would be towards requiring vaccination. There was a significant difference in attitude about vaccine policy depending on own vaccination status (Chi-square, *p* < 0.001), with vaccinated FCC providers more likely to report that they would require (13.4% of vaccinated providers versus 3.6% for unvaccinated providers) or recommend (49.1% versus 14.3%) the COVID-19 vaccine for children in their care.

Providers reported high levels of confidence in their ability to discuss both the benefits and risks of COVID-19 vaccines with the families they worked with and, the majority (>60%) somewhat-strongly) agreed that they felt confident (Table 2) in their ability to discuss both risks and benefits. There was a significant relationship between this confidence and provider vaccination status (Chi-square, *p* < 0.01 for both benefits and risks), with vaccinated providers more likely to agree that they were confident in having these conversations with families. Further, the majority of FCC providers agreed that they would recommend the vaccine to others to include the families with whom they were working.

## 4. Discussion

This research provides insight into the vaccination status, COVID-19 experience, and self-efficacy for vaccine conversations with family child care providers in Delaware as the sector continues to adapt to the pandemic. Our vaccination rate (69.8%) is similar to a national study on family child care providers fielded in May/June 2021 (73% weighted among FCC providers) [6]. More broadly, FCC provider vaccination rates were lower than the sample of center-based providers (79.7% weighted) and lower than a national sample of preschool or kindergarten teachers (81.7%) from a survey conducted on Facebook in April and May 2021 [16]. This suggests that FCC providers may have lower vaccination rates than preschool or kindergarten teachers as well. There have been limited efforts to increase vaccine uptake among FCC providers; the CDC offered a messaging toolkit for child care providers more broadly, but no specific interventions have been implemented.

Among FCC providers in Delaware, similar vaccination rates were found for FCC providers in Delaware across race and insurance status, contrary to prior findings at the national level [6]. This difference may be related to Delaware’s efforts to engage FCC providers through peer groups in communities of practice and through one-on-one technical assistance, offered in both English and Spanish. In Delaware, these efforts have been tailored to the unique strengths and needs of FCC providers since 2013 in order to enhance their quality rating and improvement system status through the Delaware Institute for Excellence in Early Childhood (DIEEC) [17]. The DIEEC has also offered FCC provider-specific communities of practice related to health education and self-care. The opportunities to interact with both DIEEC staff and other FCC providers in a manner that forge supportive relationships may have increased the trust of and credibility towards DIEEC staff promoting opportunities to receive the COVID-19 vaccine.

Our data suggest that most FCC providers feel confident in their ability to discuss both the benefits and risks of COVID-19 vaccines with the families they serve, supporting previous studies on other health topics [11]. While this study did not directly ask providers if their confidence in discussing the COVID-19 vaccine was related to encouraging or discouraging vaccination, this same block of questioning also included a question asking whether the FCC provider would recommend the vaccine to others, including families with whom they were working. Only two respondents who indicated that they would *not* recommend the vaccine also indicated that they felt confident in discussing the risks of the vaccine. It should also be noted that three providers who indicated that they would not recommend the vaccine also indicated that they felt confident in discussing the benefits. Given the low rates of COVID vaccination in children ages 5–11, and low rates of intention to vaccinate children who are younger than age five [18], the FCC providers should be considered key messengers for the families they serve and may be helpful liaisons with state and local vaccination efforts.

A recent national survey study (*N* = 682) examined primary caregiver intention to accept the COVID-19 vaccine for their children 5 years and younger when available [19]. This study suggests some hesitancy is likely and described modifiable health belief contributors to caregiver vaccine intention for their young children, such as perceived severity of COVID-19 for their child and vaccine confidence. The study also describes various demographic and mediating variables that may offer insights about caregiver decisions regarding vaccinating their young children. The results emphasize the importance of developing and disseminating proactive tailored COVID-19 vaccination education efforts for primary caregivers of young children and coupled with our study, underscores the critical role that FCC may play in this effort.

Input from FCC providers could be used to develop appropriate tailored vaccine messaging and materials that are clear, consistent, and culturally responsive for families of the children in their care and leverage knowledge gained about caregiver vaccine-related health beliefs [19]. Moreover, targeted outreach efforts to FCC providers that include training, tailored educational resources, and facilitate connections with public health officials could assist providers in sharing information about COVID vaccines with families. Supporting FCC providers in their messaging to families may be an important approach to increase vaccination rates in young children. Previous studies have explored the use of child care providers as messengers for nutrition and physical activity promotion [20] and could be a model for how such efforts could be expanded.

Strengths and Limitations: These data are self-report and prone to bias. However, one strength of the research is that these items were embedded in a larger survey about FCC well-being. This survey was conducted prior to recommendations regarding boosters, so no data is available on intentions to seek booster shots.

Future Studies: This study did not assess the demand for vaccination education in order to understand how best to tailor communication to FCC providers. An assessment on the need and desire for vaccination education among FCC providers should be part of a future investigation. In addition, vaccination status and integration of child care providers in vaccination education should be explored in other countries. It is possible that the politics and structure of the child care field in other countries have a different impact on both vaccination status and intentions of providers.

## Figures and Tables

**Table 1:** Demographics and COVID-19 vaccine status, Delaware Family Child Care Providers, 2021.

Characteristic	*n*	%
**Demographics**		
Race (*n* = 166)		
Black/African American	57	34.3
White	89	53.6
Other	20	12.0
One or more children less than 18 years of age living in the home (*n* = 163)	62	38.3
No health insurance (*n* = 161)	20	12.4
**COVID-19 experience and risk factors**		
Diagnosed with COVID-19 (*n* = 150)	13	8.7
Pre-existing condition that may put you at higher risk for COVID-19 (*n* = 150)	42	28.0
Closed at least once due to COVID-19 case in children/families (*n* = 149)	59	39.6
**COVID-19 vaccination (*n* = 149)**		
Fully vaccinated	104	69.8
Partially vaccinated	11	7.4
Of those with >1 dose (*n* = 115): first dose prior to April 2021	67	59.3
Not vaccinated	34	22.8
Intend to receive it as soon as possible ^1^	4	11.8
Intend to wait and see how it affects others in the community before I receive it ^2^	7	20.6
Do not intend on receiving it soon, but might sometime in the future ^3^	14	41.2
Do not intend to ever receive the vaccine ^4^	6	17.6
Unknown	3	8.8
**Policy towards COVID-19 vaccine for children (*n* = 140)**		
Will require vaccine	16	11.4
Will recommend	59	42.1
Will not require or recommend	31	22.1
Unsure	34	24.3

^1^ Reasons chosen by respondents (*n* = 4): Concerned about side effects (*n* = 3); do not know if vaccine will work (*n* = 1), do not like vaccines (*n* = 1). ^2^ Reasons chosen by respondents (*n* = 7): Concerned about side effects (*n* = 5); plan to wait and see if it is safe and may receive it later (*n* = 3), do not know if vaccine will work (*n* = 3), do not know enough about the vaccine yet (*n* = 3), do not like vaccines (*n* = 1), do not like needles (*n* = 1), do not trust COVID-19 vaccines (*n* = 1) ^3^ Reasons chosen by respondents (*n* = 14): Concerned about side effects (*n* = 10); do not know enough about the vaccine yet (*n* = 8), Plan to wait and see if it is safe and may receive it later (*n* = 6), do not trust COVID-19 vaccines (*n* = 6), do not know if vaccine will work (*n* = 5), do not trust the government (*n* = 4), ad COVID-19 so do not need the vaccine (*n* = 1), my doctor has not recommended it (*n* = 1), do not like needles (*n* = 1). ^4^ Reasons chosen by respondents (*n* = 6): Concerned about side effects (*n* = 5); do not trust COVID-19 vaccines (*n* = 5), do not know enough about the vaccine yet (*n* = 5), do not know if a COVID-19 vaccine will work (*n* = 2), I do not believe I need a COVID-19 vaccine (*n* = 2), I do not trust the government (*n* = 1), do not like needles (*n* = 1).

**Table 2:** Intentions and Confidence for COVID-19 vaccine conversations, Delaware Family Child Care Providers, 2021.

Statement	Strongly Disagree n (%)	Somewhat Disagree n (%)	Neither Agree Nor Disagree n (%)	Somewhat Agree n (%)	Strongly Agree n (%)
I would recommend the vaccine to others, including the families I work with.	13 (9.4)	5 (3.6)	32 (23.0)	23 (16.5)	66 (47.5)
I feel confident in my ability to discuss the benefits of COVID-19 vaccines with the families I work with.	13 (9.2)	6 (4.3)	35 (24.8)	29 (20.6)	58 (41.1)
I feel confident in my ability to discuss the risks of COVID-19 vaccines with the families I work with.	11 (7.9)	4 (2.9)	41 (29.3)	32 (22.9)	52 (37.1)

## Data Availability

The data presented in this study are available on request from the corresponding author.
